# Impaired endothelial function in siblings of patients with diabetic mellitus type 2

**DOI:** 10.1186/s40200-016-0243-9

**Published:** 2016-06-30

**Authors:** Yousef Rasmi, Kani Emamy-Nagadeh, Neda Valizadeh, Masoud Saleh-Mogadam, Alireza Shirpoor, Ehsan Saboory

**Affiliations:** 1 Cellular and Molecular Research Center, Urmia University of Medical Sciences, Urmia, Iran; 2 Department of Biochemistry, Faculty of Medicine, Urmia University of Medical Sciences, Urmia, Iran; 3 Department of Biology, Faculty of Science, Mashhad Payame-Noor University, Mashhad, Iran; 4 Department of Endocrinology, Faculty of Medicine, Urmia University of Medical Sciences, Urmia, Iran; 5 Department of Physiology, Faculty of Medicine, Urmia University of Medical Sciences, Urmia, Iran; 6 Neurophysiology Research Center, Urmia University of Medical Sciences, Urmia, Iran

**Keywords:** Siblings, Endothelial dysfunction, Diabetes mellitus, First-degree relatives

## Abstract

**Background:**

Endothelial dysfunction is considered as a risk factor for cardiovascular disease, which is a consistent finding in diabetic mellitus type 2 (DMT2). First-degree relatives of DMT2 patients have a higher risk of developing DMT2 later on the life. We aimed to investigate whether impaired endothelial function exists in siblings of DMT2 patients.

**Methods:**

As endothelial function markers, plasma E-selectin, soluble inter-cellular adhesion molecule-1 (sICAM-1), and endothelin-1 (ET-1) were measured on 27 DMT2 patients (9 m/18f; mean age: 48.48 ± 6.75 years), 28 siblings of DMT2 patients (14 m/14f; mean age: 44.54 ± 7.10 years), and 30 control subjects (18 m/12f; mean age: 44.72± 7.56 years) without any family history of diabetes. All the groups were matched by gender, age, and body mass index (BMI).

**Results:**

Plasma levels of ET-1, sICAM-1, and E-selectin were significantly higher in the DMT2 group compared to the control group (ET-1:0.79 ± 1.63 pg/ml vs. 0.33 ± 0.08 pg/ml; P_CD_ = 0.049, sICAM-1: 71.15 ± 27.20 ng/ml vs. 34.57 ± 22.56 ng/ml; P_CD_ = 0.001, E-selectin: 22.45 ± 11.57 ng/ml vs. 16.28 ± 7.50 ng/ml; P_CD_ =0.026). There was a significant difference in sICAM-1 levels between siblings (62.08 ± 26.37 ng/ml) and controls (P_CS_ = 0.002), but not between siblings and DMT2 patients (P_SD_ = 0.411). Moreover, a significant difference was observed in ET-1 levels between siblings (0.75 ± 1.26 pg/ml) and controls (P_CS_ = 0.031), but not between siblings and DMT2 patients (P_SD_ = 0.751). There was also a significant difference in E-selectin levels between DMT2 patients and siblings (16.56 ± 8.71 ng/ml; P_SD_ =0.028); however, the difference in E-selectin levels was not statistically significant between siblings and controls (P_CS_ = 0.919).

**Conclusion:**

Endothelial function markers in the siblings of DMT2 patients are increased in comparision to the control group Therefore; family history in the DMT2 patients seems to be a risk factor for endothelial function. Furthermore, endothelial dysfunction is available very early in the DMT2 patients, even before overt hyperglycemia ensues (in siblings), and may play a key role in the etiopathology of the vasculopathy associated with DMT2.

## Background

The endothelial cell lines the internal lumen of all the vasculatures and serves as an interface between circulating blood and vascular smooth muscle cells [1]. Endothelium serves as a physical barrier between the blood and tissues. Morover, it is able to respond to chemical and physical signals by production of a wide range of factors that regulate vascular tone, cellular adhesion, smooth muscle cell proliferation, and vessel wall inflammation [2].

Endothelial dysfunction has been reported in cardiovascular and metabolic disorders such as coronary heart disease [3], diabetic mellitus type I (DMT1), and diabetic mellitus type II (DMT2) [4]. The role of endothelial dysfunction is proved to be more complicated in DMT2 than in DMT1. Moreover, the effects of hyperlipidemia, aging, hypertension, and other factors add to the problem complexity [5–7]. Endothelial dysfunction and inflammation, as indicated by abnormal flow-dependent vasodilatation, increase circulating levels of adhesion molecules, soluble intercellular adhesion molecule-1 (sICAM-1), and E-selectin [8] known to occur in patients with DMT2 [9, 10].

E-selectin is a cell adhesion molecule expressed only on endothelial cells activated by cytokines. Like other selectins, it plays an important part in inflammation [11]. During inflammation, E-selectin plays an important role in recruiting leukocytes to the site of injury. The local release of cytokines IL-1 and TNF-α by damaged cells induces the over-expression of E-selectin on endothelial cells of nearby blood vessels [12].

ICAM-1 is a member of the immunoglobulin superfamily. Moreover, ICAM-1 is regarded as a transmembrane protein possessing an amino-terminus extracellular domain, a single transmembrane domain, and a carboxy-terminus cytoplasmic domain [13].

On the other hand, endothelin-1 (ET-1), a potent vasoconstrictor peptide, is potentially involved in the vasomotor dysregulation of patients with diabetes as well as in the development of their vascular complications [14, 15]. Further, both hyperglycaemia and insulin administration elevate circulating levels of ET-1 [9, 10]. As ET-1 plays a pathophysiologic role in various forms of cardiovascular disease, it has been suggested to be a potential factor in endothelial dysfunction [16].

In fact, markers of endothelial function are often elevated years before any sign of microangiopathy becomes evident [5–7]. First-degree relatives (FDR) of DMT2 patients are at increased risk of coronary artery disease and also have a lifetime risk of developing DMT2 of up to 40 % [17]. Development of these dysfunctions appears to be predicted by the presence of features of insulin resistance and associated clustering of atherosclerotic risk in these subjects [18]. In addition to the insulin resistance found in young FDRs [19, 20], family studies have revealed that FDRs of individuals with DMT2 are about 3 times more likely to develop DMT2 than individuals without a positive family history of the disease [21, 22].

This research aimed to assess endothelial function in FDRs and compare it with that in DMT2 and normal subjects. Moreover, considering that diabetic patients display altered endothelial function, we designed the present study so that circulating levels of endothelial function markers could be assessed in patients with DMT2 and their siblings.

## Methods

### Subjects

The participants in this study included 27 patients with DMT2, 28 siblings of DMT2 patients, and 30 control subjects with an age range from 30–60 years. Non-smoker patients who had DMT2 after the age of 30 were only classified as having DMT2 and therefore, included in the study. Patients were then asked to encourage their siblings to participate in the study and the significance of the study was clarified to those willing to participate. Further, siblings of type 2 diabetic patients, matched for age, gender and body mass index (BMI) were studied.

Control subjects without personal or close family history of DMT2 were then selected to be matched with the DMT2 patients and their siblings in terms of age, gender and BMI. This study was conducted in accordance with the Declaration of Helsinki [23], and its revised version was ultimately accepted by the Ethics Committee of UMSU, Urmia, Iran. All the patients were required to give written informed consent prior to participating in the study.

### Blood specimens

Peripheral heparinized blood samples (5 ml) were collected from the subjects in the morning after an overnight fasting in clean glass tubes and then centrifuged at 3000 rpm for 6 min. The plasma was then separated and stored at−80 °C until analysis.

### Assessment of endothelial function markers and fasting blood sugar (FBS)

All the obtained samples were subjected to the following investigations: 1) Determining plasma concentrations in sICAM-1, ET-1, and sE-selectin using enzyme-linked immunosorbent assay kits (IBL; North America), 2) Determining plasma fasting blood sugar according to the glucose oxidase method (ZistChemi Diagnostics; Iran) using an autoanalyzer.

### Exclusion criteria

Subjects were carefully selected to exclude conditions that could interfere with endothelial dysfunction markers, including coronary heart disease, smoking, hypertension, bronchial asthma, acute or chronic inflammatory diseases, autoimmune diseases, and medications like steroids and antipsychotic drugs.

### Statistical analysis

Mean and *P* values were estimated using SPSS (version 18). Results are expressed as mean ± SD. Moreover, multiple comparison tests were performed using ANOVA, followed by LSD and post-hoc analysis to locate any differences. A *P*-value less than 0.05 indicated statistical significance.

## Results

The demographic and clinical characteristics of the experimental population are listed in Table 1. Twenty-seven patients with DMT2 (33.3 % male), 28 siblings of DMT2 patients (50 % male), and 30 control individuals (60 % male) were studied. Duration of disease in the DMT2 patients was 92.89 ± 67.97 month. The results showed significant differences in fasting blood sugar levels in the DMT2 patients (187.08 ± 50.79 mg/dl) compared to those in the siblings (89.25 ± 7.69 mg/dl) and the controls (82.99 ± 6.46 mg/dl) (*p* < 0.0001, for both). However, the difference in plasma glucose levels between the siblings and the control individuals was not statistically significant (*p* = 0.452), as shown in Table 1.

**Table 1:** Clinical and laboratory characteristics of study participants

Variable	Control	Siblings	DMT2	P_CS_	P_CD_	P_SD_
Age (year)	44.72 ± 7.56	44.54 ± 7.10	48.48 ± 6.75	0.99	0.17	0.11
Sex (M/F)	18/12	14/14	9/18	0.44	0.10	0.21
BMI (kg/m^2^)	28.81 ± 4.51	30.64 ± 5.87	28.65 ± 3.84	0.17	0.96	0.13
Systolic blood pressure (mm Hg)	131.4 ± 07.6	140.8 ± 15.1	145.1 ± 11.7	0.006	<0.001	0.19
Diastolic blood pressure (mm Hg)	88.1 ± 09.9	117.6 ± 147.7	93.7 ± 11.9	0.22	0.81	0.31
Disease period (month)	0	0	92.89 ± 67.97	-	-	-
Fasting blood sugar (mg/dl)	82.99 ± 6.46	89.25 ± 7.69	187.08 ± 50.79	0.452	<0.001	<0.001

*DMT*2 diabetes mellitus type 2, *P*_*CS*_ *P* value between controls and sibling, *P*_*CD*_ *P* value between controls and DMT2, *P*_*SD*_ *P* value between DMT2 and sibling

As shown in Table 2, plasma levels of ET-1 were significantly higher in the DMT2 group than in the control group (0.79 ± 1.63 pg/ml vs. 0.33 ± 0.08 pg/ml, respectively; P_CD_ = 0.049). In addition, there were significant differences in ET-1 levels between the siblings (0.75 ± 1.26 pg/ml) and the controls (P_CS_ = 0.031), but not between the siblings and the DMT2 patients (P_SD_ = 0.751).

**Table 2:** Levels of endothelial function variables in DMT2, Sibling and controls

Variable	Control	Siblings	DMT2	P_CS_	P_CD_	P_SD_
ET-1 (pg/ml)	0.33 ± 0.08	0.75 ± 1.26	0.79 ± 1.63	0.031	0.049	0.751
sICAM-1 (ng/ml)	34.57 ± 22.56	62.08 ± 26.37	71.15 ± 27.2	0.002	0.001	0.411
sE-selectin (ng/ml)	16.28 ± 7.50	16.56 ± 8.71	22.45 ± 11.57	0.919	0.026	0.028

*DMT*2 diabetes mellitus type 2, *P*_*CS*_ *P* value between controls and siblings, *P*_*CD*_ *P* value between controls and DMT2, *P*_*SD*_ *P* value between DMT2 and siblings

Plasma sICAM-1 levels were similar in the DMT2 patients (71.15 ± 27.20 ng/ml) and the siblings (62.08 ± 26.37 ng/ml; P_SD_ = 0.411), but lower in the controls (34.57 ± 22.56 ng/ml; P_CD_ = 0.001). Data analysis also revealed a significant difference in sICAM-1 levels between the siblings and the control subjects (P_CS_ = 0.002), and between the DMT2 patients and the control subjects (P_CD_ = 0.001), as indicated in Table 2. Levels of endothelial expression of sE-selectin were higher in the DMT2 subjects (22.45 ± 11.57 ng/ml) than in the siblings (16.56 ± 8.71 ng/ml) and the control (16.28 ± 7.50 ng/ml) subjects (P_SD_ = 0.028 and P_CD_ = 0.026, respectively). Furthermore, no significant differences in sE-selectin concentrations were observed between the two groups of siblings and controls (P_CS_ = 0.919).

## Discussion

Researchers have reported that FDRs of DMT2 patients are more likely to show impaired endothelial function in the resistance vessels than those with no family history of the condition [24]. Ostergård et al. showed that healthy, but insulin-resistant FDRs of DMT2 subjects predisposed for DMT2 show minor signs of endothelial dysfunction [25]. More recently, Caballero et al. [26] demonstrated early abnormalities in vascular reactivity and biochemical markers of endothelial cell activation in individuals at risk of developing DMT2. Cellular adhesion molecules are poorly expressed by the resting endothelium, but are up-regulated during inflammatory atherogenesis and may be an index of endothelial activation or even a molecular of early atherosclerosis [27]. The results indicated that the concentrations of ET-1 and sICAM-1 were significantly higher in the FDRs of DMT2 subjects than in the control subjects. In patients with DMT2, endothelial cell dysfunction is detectable very early in the course of the disease, even before overt hyperglycemia ensues, and may play a key function in the etiopathology of the vasculopathy reported with DMT2. The expression of VCAM-1, sICAM-1, and sE-selectin plays a role in the initiation of the inflammatory process [28]. However, only a few studies have examined the development and progression of endothelial dysfunction in siblings of DMT2 patients. We found a highly significant plasma level of ET-1 and sICAM-1 in the siblings of DMT2 subjects. It is also possible that some genetic abnormalities leading to endothelial dysfunction could be present in individuals with family history of DMT2, although another study found that only those FDRs with demonstrable insulin resistance had endothelial dysfunction [29]. We have demonstrated that FDRs of DMT2 patients involve factor (s) capable of inducing the expression of ET-1 and sICAM-1 in endothelial cells. This effect cannot be directly attributed to hyperglycemia. Increased expression of ET-1 and sICAM-1 may have a role in the development of endothelial dysfunction in the siblings of DMT2 subjects. The present study also shows that Et-1 and sICAM-1 are sensitive markers of endothelial activation, in contrast to sE-selectin. In a study by Dai Wu et al. [30], the levels of E-selectin, vascular cell adhesion molecule-1 (VCAM-1), total cholesterol, and triglyceride were significantly elevated in FDR group compared with controls. There were endothelial dysfunction, activation of adhesion molecule, and insulin resistance in FDRs, and endothelial dysfunction is positively related to insulin resistance. Similarly, Gómez et al. designed another study in order to demonstrate whether FDRs of DMT2 subjects present markers as a form of precocious indicators of diabetes mellitus. They showed ICAM-1 and VCAM-1 to be significantly higher in the diabetic group, but not in the family group [31]. Our study should be interpreted within the context of its possible limitations such as low number of subjects in the groups.

## Conclusions

We showed that the subjects with DMT2 have higher ET-1 and sICAM-1 levels than the controls. Moreover, significant increase of ET-1 and sICAM-1 in the siblings showed the high ability of these markers in evaluating endothelial function. In addition, sICAM-1 and sE-selectin levels correlated with blood glucose. This finding confirms the importance of glycaemic milieu in determining circulating levels of endothelial function markers.

## Abbreviations

DMT2, Diabetic mellitus type 2; ET-1, Endothelin-1; FDR, First-degree relative; sICAM-1, Soluble inter- cellular adhesion molecule-1
